# Cranial and extracranial manifestations of giant cell arteritis: a single-center observational study

**DOI:** 10.1007/s00296-024-05608-2

**Published:** 2024-05-13

**Authors:** Emilia Kudraszew, Anna Nowakowska-Płaza, Jakub Wroński, Mateusz Płaza, Małgorzata Wisłowska

**Affiliations:** 1 Department of Rheumatology, National Institute of Geriatrics, Rheumatology and Rehabilitation, Spartańska 1, Warsaw, 02-637 Poland; 2 Department of Radiology, National Institute of Geriatrics, Rheumatology and Rehabilitation, Warsaw, Poland

**Keywords:** Giant cell arteritis, Cranial, Extracranial, Diagnosis, Criteria

## Abstract

**Introduction:**

Giant cell arteritis (GCA) presents two major phenotypes – cranial (cGCA) and extracranial (exGCA). exGCA may be overlooked. The study aimed to compare the clinical characteristics between cGCA and exGCA.

**Methods:**

Electronic medical records of patients treated between January 2015 and July 2023 at the Department of Rheumatology were searched for the diagnosis of GCA. The clinical characteristics of patients with cGCA, exGCA, and overlapping GCA manifestations were compared.

**Results:**

Out of 32 patients with GCA, 20 had cGCA, 7 had exGCA, and 5 had overlap manifestations. The groups did not differ significantly in demographics, clinical signs/symptoms, or laboratory test results. Importantly, the combined group of patients with exGCA and overlap GCA had a statistically significant delay in initiating treatment (median 12 weeks) compared to patients with cGCA (median 4 weeks; *p* = 0.008).

**Conclusion:**

Our study confirmed the insidious nature of exGCA, which lacks distinctive clinical symptoms and consequently leads to delayed treatment.

**Supplementary Information:**

The online version contains supplementary material available at 10.1007/s00296-024-05608-2.

## Introduction

Giant cell arteritis (GCA) belongs to the group of large vessel vasculitis according to the 2012 International Chapel Hill Consensus Conference nomenclature [1]. The pooled incidence of GCA is 10 cases per 100,000 individuals aged 50 years and older [2]. Due to the aging population, the number of patients affected by GCA is expected to rise. The systematic literature review by de Smit et al. predicts that by 2050, more than 3 million people will be diagnosed with GCA, resulting in visual impairment for 500,000 individuals all over the world [3].

Although the inflammatory process in GCA affects different large vessels, GCA is historically associated only with inflammation of the temporal artery (TA). American College of Rheumatology (ACR) classification criteria 1990 [4] included clinical symptoms related only to the skull (headache, TA tenderness) and the histopathological result of the biopsy of TA. The need to classify patients with diseases affecting not exclusively cranial arteries was one of the reasons for creating the new ACR/European Alliance of Associations for Rheumatology (EULAR) GCA classification criteria published in December 2022 [5]. Clinically, there are two phenotypes of the disease: cranial (cGCA), primarily associated with the temporal artery or other branches of the external carotid arteries, and extracranial (exGCA), characterized by inflammation of large arteries such as the aorta and its branches [6, 7]. Although exGCA may resemble another large vessel vasculitis, Takayasu’s disease, the main differences are the older age of the patients (typically over 50 years of age), the co-occurrence of polymyalgia rheumatica (PMR) in patients with exGCA, and the absence of involvement of sub-diaphragmatic arteries. In clinical practice, cGCA is the most common form of GCA [8]. Both manifestations may overlap. There is no clear data on the incidence of exGCA. Literature data estimating the incidence of exGCA to range from 3% (in studies that included diagnosis only based on clinical symptoms) to 83% (in a study where all patients underwent fluorodeoxyglucose positron emission tomography-computed tomography - FDG PET-CT) [6]. Solitary exGCA (without cGCA) occurs the least frequently and often presents with non-specific signs and symptoms, presenting a diagnostic challenge.

Due to the insidious nature of exGCA symptoms, exGCA may be overlooked. In our study, we aimed to compare the clinical characteristics of GCA patients with cranial and extracranial involvement.

## Patients and methods

Electronic medical records of patients admitted to the Department of Rheumatology between January 2015 and July 2023 were retrospectively searched for the diagnosis of GCA (ICD-10 code M31.5, M31.6). Ethics approval for this study was waived by the Institutional Bioethics Committee resolution (number KBT-1/2/2018) due to the retrospective nature of the medical record analysis. The diagnosis of GCA was established in 32 patients based on the clinical presentation, laboratory and imaging tests, and considering the ACR 1990 classification criteria [4]. We retrospectively reevaluated patients for meeting ACR/EULAR 2022 classification criteria [5].

Data regarding patient characteristics (sex, age, BMI, and cardiovascular comorbidities), clinical signs and symptoms (vision loss, scalp tenderness, temporal headache, jaw/tongue/limbs claudication, PMR symptoms, fever, weight loss, abnormal physical examination of TA, and anterior ischemic optic neuropathy [AION] in ophthalmological examination), laboratory (levels of C-reactive protein [CRP] and erythrocyte sedimentation rate [ESR]) and imaging test performed, and time of initiation of immunosuppressive treatment were collected. We applied the same cut-off values for the elevation of inflammatory markers as specified in ACR/EULAR 2022 classification criteria [5]: CRP ≥ 10 mg/L and ESR ≥ 50 mm/h. Imaging tests were performed based on clinical indications at the discretion of the attending physician. Imaging tests included color Doppler ultrasonography (CDU; with probes ≥ 15 MHz for cranial arteries and ≥ 7 MHz for the other arteries, according to EULAR recommendations for the use of imaging in large vessel vasculitis [9]) and computed tomography angiography (CTA; Siemens Somatom Definition AS 128-slices, contrast agent: ultravist). CDU of the temporal arteries was conducted in 30 patients, carotid and vertebral in 27 patients, and axillary arteries in 14 patients. Vessels were assessed for GCA involvement in CDU based on the presence of hypoechoic thickening of the intima-media not responding to pressure (“halo sign”), according to cut-off values presented by Schmidt et al. [10], stenosis, or occlusion. The CTA of large vessels (carotid and vertebral arteries, thoracic and abdominal aorta, and aorta branches) was performed in 14 patients, with evaluation for vessel wall thickening, aneurysm/ectasia, or stenosis. The patient medical records also included the results of tests performed outside our hospital - in 3 patients the FDG PET-CT and in 1 patient TA biopsy results (in our center, biopsies are not performed for technical reasons).

### Statistical analysis

For statistical analysis, patients were categorized into three groups based on imaging tests and histopathological findings: the cGCA group (with exclusively cranial vessel involvement), exGCA (with isolated large vessel involvement), and the overlap group (overlapGCA; with both cranial and extracranial vessel involvement). In the case of GCA diagnosis without confirmed vascular involvement in imaging studies or biopsy, patients were assigned to a specific group based on the presented clinical signs and symptoms. An additional analysis was performed to compare patients with cGCA and patients with large vessel involvement, regardless of the presence of cranial involvement (combined-exGCA).

Compliance of the data with the normal distribution was assessed using the Shapiro-Wilk test. For variables demonstrating normal distribution differences between multiple groups were assessed with ANOVA with post hoc analysis via the Bonferroni test and between two groups with Student’s t-test. For variables without normal distribution, the Kruska-Wallis test with post hoc analysis using the Dunn’s test and Mann-Whitney U test were applied, respectively. For categorical variables, Fisher’s exact test was employed (due to tables containing values less than 5; subsequent pairwise testing was conducted with the 2 × 3 test). Statistical analysis was performed using Statistica 13.3 software.

## Results

Throughout the study period, 32 patients (23 females, 9 males) were diagnosed with GCA in our rheumatology department – 20 (62.5%) had cGCA, 7 exGCA (21.9%), and 5 overlapGCA (15.6%). The characteristics of the study cohorts are presented in Table 1.

**Table 1:** Characteristics of GCA patients with cranial, extracranial, and overlap involvement. AION – anterior ischemic optic neuropathy in an ophthalmological examination, BMI – body mass index, CRP – C-reactive protein, CT – computed tomography, ESR – erythrocyte sedimentation rate, FDG-PET – fluorodeoxyglucose positron emission tomography-computed tomography, n – number, TA – temporal artery, US – ultrasound. * *p* < 0.05 compared to the extracranial group

Demographics				
	All patients (*n* = 32)	Cranial (*n* = 20)	Extracranial (*n* = 7)	Overlap (*n* = 5)
Females, n (%)	23 (71.9%)	12 (60%)	7 (100%)	4 (80%)
Age, mean (± SD)	71.8 (± 8)	72.2 (± 7.6)	71.3 (± 10)	70.8 (± 8.7)
Hypertension, n (%)	26 (81.3%)	16 (80%)	7 (100%)	3 (60%)
Coronary artery disease, n (%)	10 (31.3%)	6 (30%)	2 (28.6%)	2 (40%)
	All patients (*n* = 25)	Cranial (*n* = 16)	Extracranial (*n* = 5)	Overlap (*n* = 4)
BMI, mean (± SD)	26.8 (± 5.4)	27.5 (± 5.9)	25 (± 4.8)	26.3 (± 4.6)
Signs and symptoms				
	All patients (*n* = 32)	Cranial (*n* = 20)	Extracranial (*n* = 7)	Overlap (*n* = 5)
Sudden vision loss, n (%)	14 (43.8%)	11 (55%)	1 (14.3%)	2 (40%)
AION, n (%)	9 (28.1%)	8 (40%)	1 (14.3%)	0 (0%)
AION, bilateral n (%)	4 (12.5%)	3 (15%)	1 (14.3%)	0 (0%)
Blindness, n (%)	4 (12.5%)	3 (15%)	1 (14.3%)	0 (0%)
Abnormal examination of TA, n (%)	17 (53.1%)	12 (60%)	2 (28.6%)	3 (60%)
Fever, n (%)	10 (31.3%)	5 (25%)	4 (57.1%)	1 (20%)
Weight loss > 5 kg, n (%)	3 (9.4%)	1 (5%)	1 (14.3%)	1 (20%)
Scalp tenderness, n (%)	7 (21.9%)	5 (25%)	2 (28.6%)	0 (%)
New temporal headache, n (%)	23 (71.9%)	13 (65%)	5 (71.4%)	5 (100%)
Jaw/tongue claudication, n (%)	8 (25%)	5 (25%)	1 (14.3%)	2 (40%)
Limb claudication, n (%)	4 (12.5%)	3 (15%)	0 (0%)	1 (20%)
Polymyalgia symptoms, n (%)	12 (37.5%)	8 (40%)	2 (28.6%)	2 (40%)
Laboratory tests				
ESR before treatment	All patients (*n* = 28)	Cranial (*n* = 16)	Extracranial (*n* = 7)	Overlap (*n* = 5)
- ≥50 mm/hour, n (%)	22 (78.6%)	12 (75%)	6 (85.7%)	4 (80%)
- mean (± SD)	74.6 (± 30.7)	74.1 (± 32.1)	69.4 (± 30.5)	83.8 (± 30.8)
CRP	All patients (*n* = 24)	Cranial (*n* = 13)	Extracranial (*n* = 6)	Overlap (*n* = 5)
- ≥10 mg/L, n (%)	24 (100%)	13 (100%)	6 (100%)	5 (100%)
- mean (± SD)	96.3 (± 63.6)	107.9 (± 62.3)	73.9 (± 46.2)	92.8 (± 88)
Imaging tests				
TA US	All patients (*n* = 30)	Cranial (*n* = 20)	Extracranial (*n* = 5)	Overlap (*n* = 5)
Halo sign, n (%)	21 (70%)	16 (80%)	0 (0%)	5 (100%)
Halo sign, bilateral n (%)	12 (40%)	9 (45%)	0 (0%)	3 (60%)
	All patients (*n* = 27)	Cranial (*n* = 19)	Extracranial (*n* = 4)	Overlap (*n* = 4)
Intima-media thickness, mm median (range)	0.53 (0.2–1.25)	0.6 (0.2-1)*	0.28 (0.25–0.29)	0.48 (0.43–1.25)
US/CT/FDG-PET carotid and vertebral arteries assessment	All patients (*n* = 27)	Cranial (*n* = 15)	Extracranial (*n* = 7)	Overlap (*n* = 5)
Carotid arteries involvement, n (%)	6 (18.8%)	0 (0%)	3 (42.9%)	3 (60%)
Vertebral arteries involvement, n (%)	3 (11.1%)	0 (0%)	2 (28.6%)	1 (20%)
US/CT/FDG-PET axillary arteries assessment	All patients (*n* = 21)	Cranial (*n* = 11)	Extracranial (*n* = 6)	Overlap (*n* = 4)
Axillary involvement, n (%)	3 (14.3%)	0 (0%)	2 (33.3%)	1 (20%)
Axillary involvement, bilateral n (%)	3 (14.3%)	0 (0%)	2 (33.3%)	1 (20%)
CT/FDG-PET aorta assessment	All patients (*n* = 15)	Cranial (*n* = 6)	Extracranial (*n* = 6)	Overlap (*n* = 3)
Aorta involvement, n (%)	9 (60%)	0 (0%)	6 (100%)	3 (100%)
FDG-PET aorta assessment	All patients (*n* = 3)	Cranial (*n* = 0)	Extracranial (*n* = 2)	Overlap (*n* = 1)
Aorta involvement in FDG-PET, n (%)	3 (14.3%)	-	2 (100%)	1 (100%)
Classification criteria				
	All patients (*n* = 32)	Cranial (*n* = 20)	Extracranial (*n* = 7)	Overlap (*n* = 5)
Meet ACR 1990 criteria	23 (71.9%)	14 (71.4%)	5 (71.4%)	4 (80%)
Meet ACR/EULAR 2022 criteria	29 (90.6%)	20 (100%)	4 (57.1%)	5 (100%)

We found no significant differences between groups in sex, age, BMI, and cardiovascular comorbidities. In the cGCA group, the most common signs/symptoms were new temporal headache (65%), abnormal examination of TA (60%), and sudden vision loss (55%). In the exGCA group the most common signs/symptoms were new temporal headache (71.4%), fever (57.1%), scalp tenderness (28.6%), and PMR symptoms (28.6%). In the overlap group, the most common signs/symptoms were new temporal headache (100%), abnormal examination of TA (60%), PMR symptoms (40%), jaw/tongue claudication (40%), and sudden vision loss (40%). Nevertheless, no statistically significant differences were found in signs/symptoms nor inflammatory markers between the groups. The differences also did not reach statistical significance when comparing cGCA with combined-exGCA. Differences between groups, according to the adopted division, were observed in the results of imaging tests. The thickness of the intima-media of TA was significantly greater in cGCA than in exGCA (mean 0.6 mm vs. 0.28 mm, *p* < 0.019), and the halo sign was present in 80% of cGCA patients. Among the extracranial vessels (in patients who underwent imaging of a given body region), the most frequently involved vessels were the aorta (60%), carotid arteries (18.8%), axillary arteries (14.3%), and vertebral arteries (11.1%).

Notably, a statistically significant delay in initiating the treatment was observed in patients with combined-exGCA (median 12, range 1–24 weeks) compared to the patients with cGCA (median 4, range 4–28 weeks; *P* = 0.008). However, when comparing individual groups (exGCA and overlapGCA separately), the difference in treatment delay did not reach statistical significance.

Among all patients diagnosed with GCA, 71.9% met the ACR 1990 classification criteria, while 90.6% the ACR/EULAR 2022 classification criteria. Statistically significantly more patients with cGCA met the new criteria (100% vs. 71.4%; *P* = 0.021), but a comparable number of patients with exGCA (57.1% vs. 71.4%) and overlapGCA (100% vs. 80%).

## Discussion

In our study, we found that 21.9% of our patients had exGCA, which is consistent with literature data [6]. We observed that none of the clinical signs and symptoms were specific to a particular phenotype of GCA. However, non-specific systemic symptoms, such as fever and PMR symptoms, were among the most common in exGCA. This is consistent with a series of studies from Spain in which the authors demonstrated a higher incidence of PMR [11–13] and fever [12] among patients with exGCA compared to cGCA. This indicates the need to pay attention in everyday clinical practice to the general symptoms of GCA. It’s noteworthy, as GCA may manifest solely as a fever of unknown origin [14]. One study also showed that patients with exGCA may experience fewer headaches compared to those with cGCA [15], although this finding has not been confirmed by any of the other studies [13, 16]. Additionally, certain studies noted differences in the incidence of visual disturbances/AION, which were more frequent in cGCA [11, 12] or overlap GCA [13] compared to exGCA. In our GCA group, among the most frequently observed symptoms were vision loss and AION. This is consistent with literature data according to which the incidence of AION ranges from 6.3 to 48% [17]. Although subjects with exGCA and overlap GCA had less AION compared to cGCA, this difference did not reach statistical significance (probably due to the small sample size, similar to other studies [15, 16]). Finally, one study reported that patients with cGCA were older than those with exGCA [11]. However, this observed age difference was not confirmed in other studies, including ours [12, 13, 15, 16].

Our study also did not demonstrate differences in inflammatory marker levels between cGCA and exGCA. There are discrepancies in the literature on this topic. Although no studies have found a difference between CRP levels between cGCA and exGCA patients, some studies indicate that cGCA patients may exhibit higher ESR levels [11, 12, 18]. It is worth noting, however, that elevated ESR may be a less sensitive marker than elevated CRP. In our study, while all patients had CRP above 10 mg/L, 21% of patients did not surpass an ESR above 50 mm/h. This is consistent with literature data indicating that up to 22.5% of patients with GCA may not have elevated ESR [19].

These discrepancies among studies may stem from the lack of a commonly accepted phenotype classification of GCA patients. Some studies report patients with extracranial vascular involvement regardless of cranial involvement as exGCA [15], whereas other studies divide patients into overlap and isolated extracranial involvement [12, 13, 16], or only report isolated exGCA [11]. Additionally, there is no consensus regarding the criteria for assessing vascular involvement - some authors divide patients according to signs and symptoms [11, 15], while others do not describe the selected selection methodology [12, 16]. In our study, we decided to rely on the results of imaging tests (following the approach of Monjo et al. [13]), as they offer greater objectivity compared to signs and symptoms. However, given imaging tests’ limited sensitivity, this could result in misclassification of patients. In our study, one patient presented symptoms of AION, but had changes in imaging tests only in extracranial vessels, and therefore was classified as exGCA. Without adopting a universally accepted division of phonotypes of GCA patients, comparing the results of different studies will be difficult.

Currently, only imaging tests can help detect exGCA [6, 7]. Among the imaging tests, CDU is recommended to be the first-line examination in patients with suspected GCA [20, 21]. This recommendation works in practice not only for TA (as evidenced by the halo sign observed in 80% of cGCA patients in our study) but also for extracranial vessels involvement. Specifically, 75% of our patients with combined-exGCA exhibited large vessels involvement in CDU examination. This is even more important, as access to imaging techniques superior to CDU for imaging large vessel involvement (CTA, MRI, FDG PET-CT) may be subject to local availability. In our study only 3 patients had FDG PET-CT performed, as in Poland, access to the FDG PET-CT is difficult due to reimbursement indications for the test. It should be emphasized, however, that although CDU is not able to assess the aorta and its branches, a recent report indicates that a negative US examination for extracranial involvement suggests a low risk of aortitis. In a study of 72 patients with GCA who underwent both CDU and PET-CT, only 2 patients with aortitis had no changes in other extracranial vessels on CDU [22].

Confirmation of GCA diagnosis should not delay the initiation of treatment following the EULAR 2023 recommendations [21]. Therefore, it is concerning that our findings indicate a delay in treatment initiation for exGCA (12 weeks vs. 4 weeks). This is consistent with the results of a systematic review and meta-analysis, which showed a delay of 17.6 weeks in the diagnosis of exGCA compared to 7.7 weeks in cGCA [23]. This fact indicates the need for greater awareness of the existence of exGCA among clinicians.

The new ACR/EULAR classification criteria published in 2022 embraced the existence of the exGCA by including extracranial features in the criteria [5]. However, a recent study evaluating the performance of the new 2022 ACR/EULAR classification criteria across different GCA types showed lower sensitivity of the criteria in the exGCA group compared to cGCA [24]. What’s more, the criteria alone may not be sufficient to improve the diagnostic sensitivity of exGCA, unless they are supported by routine imaging for extracranial vascular involvement in all patients. In our cohort, the new ACR/EULAR criteria did not aid in the diagnosis of exGCA.

The strength of our study lies in its comparison of multiple clinical, laboratory, and imaging factors between patients with cGCA and exGCA. However, our study also has its limitations. The major limitation is the small sample size (resulting from the rare nature of the disease itself) from the single-center and retrospective character of the study (resulting in missing data). This may have prevented certain differences between cGCA and exGCA from attaining statistical significance. Moreover, our study is susceptible to selection bias, as imaging was conducted at the physician’s discretion rather than according to a standardized protocol. The fact that not all patients had imaging for extracranial vessel involvement may have resulted in an underestimation of the size of the exGCA group.

## Conclusions

Our study confirmed the insidious nature of exGCA, which may lack distinctive clinical symptoms and consequently result in delayed treatment. It highlighted the pivotal role of imaging tests, including the cost-effective and readily available CDU test, not only in diagnosing cGCA but also in identifying cases of exGCA.

### Electronic supplementary material

Below is the link to the electronic supplementary material.

Supplementary Material 1

## Data Availability

Available upon reasonable request sent to the corresponding author.
